# *Amaranthus spinosus* Linn. Extract as an Innovative Strategy to Regulate Biomarkers for Ovarian Hyperthecosis via Circular RNA (hsa‐circ‐0001577): Evidence From Biochemical, Metabolomics, Histological, and Phytochemical Profiling

**DOI:** 10.1002/fsn3.70314

**Published:** 2025-05-19

**Authors:** Naglaa M. Ammar, Mai O. Kadry, Asmaa S. Abd Elkarim, Reham S. Ibrahim, Ibrahim E. Sallam, Abd El‐Nasser G. El Gendy, Sherif M. Afifi, Tuba Esatbeyoglu, Mohamed A. Farag, Abdelsamed I. Elshamy

**Affiliations:** ^1^ Therapeutic Chemistry Department National Research Centre Giza Egypt; ^2^ Chemistry of Tanning Materials and Leather Technology Department National Research Centre Giza Egypt; ^3^ Department of Pharmacognosy, Faculty of Pharmacy Alexandria University Alexandria Egypt; ^4^ Pharmacognosy Department, College of Pharmacy October University for Modern Sciences and Arts (MSA) Giza Egypt; ^5^ Medicinal and Aromatic Plants Research Department National Research Centre Giza Egypt; ^6^ Department for Life Quality Studies University of Bologna Rimini Italy; ^7^ Department of Molecular Food Chemistry and Food Development, Institute of Food and One Health Gottfried Wilhelm Leibniz University Hannover Hannover Germany; ^8^ Pharmacognosy Department, Faculty of Pharmacy Cairo University Cairo Egypt; ^9^ Department of Natural Compounds Chemistry National Research Centre Giza Egypt

**Keywords:** *Amaranthus spinosus*, circular RNAs, metabolomics, ovarian hyperthecosis, phytochemical profile

## Abstract

*Amaranthus* species, including *A. spinosus* Linn, are well‐known vegetables whose leaves, shoots, fragile stems, and grains are commonly utilized as herbs in soups or sauces, aside from traditional uses to treat a wide range of illnesses. Ovarian hyperthecosis is a common syndrome associated with metabolomics and endocrinology that lowers female fertility. The investigation of novel biomarkers and targeted therapies for the detection and treatment of ovarian hyperthecosis is of interest. Types of noncoding RNAs known as circular RNAs (circRNAs) have covalently closed cyclic structures, are widely distributed, and exhibit expression patterns that are particular to different stages of development. Ovarian hyperthecosis was induced in rats via dehydroepiandrosterone (DHEA) followed by 1 month of treatment with 50 and 100 mg/kg of the *A. spinosus* EtOH extract. Further, oxidative stress biomarkers including GSH and MDA were investigated in addition to hormonal biomarkers, such as Luteinizing hormone and testosterone hormone, a metabolomics approach modeled using orthogonal partial least squares discriminant analysis (OPLS‐DA), and circRNA (hsa‐circ‐0001577). Furthermore, UHPLC‐ESI‐Orbitrap‐MS analysis was used for metabolites profiling to identify active agents in the plant extract. Results revealed a significant improvement in these biomarkers in the DHEA group treated with *A. spinosus* , especially at high doses, and further confirmed via histopathological assays. Multivariate data analyses of serum metabolome indicated significant variations in serum profiles among normal, disease, and treated groups. Variable importance in the projection (VIP) values guided the selection of differentiated metabolites, revealing significant changes in metabolite concentrations. UHPLC‐ESI‐Orbitrap‐MS analysis identified 72 bioactive metabolites belonging to phenolics, triterpenoidal saponins, and pyridines In conclusion, *A. spinosus* could be a management approach for ovarian hyperthecosis therapy via regulating circRNA (hsa‐circ‐0001577), disturbed hormonal balance, and metabolomics biomarkers based assays.

## Graphical abstract

A significant improvement in hormonal and oxidative stress biomarkers was observed in rats treated with *Amaranthus spinosus* , especially at high doses, after induction of ovarian hyperthecosis. *Amaranthus spinosus* could be a management approach for ovarian hyperthecosis via regulating circRNA (hsa‐circ‐0001577), disturbed hormonal balance, and metabolomics biomarkers based assays.

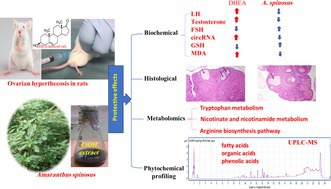

## 1 Introduction

Ovarian hyperthecosis is a prevalent hormonal disorder affecting women of reproductive age. Hormonal abnormalities, irregular periods, high testosterone, and ovarian cysts can all be symptoms of ovarian hyperthecosis (Jing et al. 2023). Six to 20% of reproductive‐age women have ovarian hyperthecosis, a very common endocrinologic disease (Rosenfield and Ehrmann 2016). Hyperandrogenism, polycystic ovarian morphology, and irregular menstruation are some of the characteristics of that disorder (Goodarzi et al. 2011). Additionally, metabolic conditions, such as insulin resistance, obesity, hyperlipidemia, type 2 diabetes mellitus, and cardiovascular disease are linked to ovarian hyperthecosis (Goodarzi et al. 2011). Ovarian hyperthecosis is linked to pregnancy‐related complications, including gestational diabetes, antepartum hemorrhage, preterm birth, and pregnancy‐induced hypertension (Palomba et al. 2015). It is difficult to diagnose and treat ovarian hyperthecosis because its etiology and pathophysiology are still unknown. Determining the underlying causes of ovarian hyperthecosis, investigating its clinical markers, and creating more potent therapies are therefore essential.

Genetic variables and epigenetic changes are linked to the complex onset and progression of ovarian hyperthecosis (Ajmal et al. 2019). Noncoding RNAs (ncRNAs), which are important elements of the epigenetic regulatory network, have been shown to contribute to the development of ovarian hyperthecosis, offering novel possibilities for diagnosis, prognosis, and treatment (Mu et al. 2021). Circular RNAs (circRNAs) are a new type of endogenous ncRNA with a covalently closed structure. They are primarily produced by the transcription of protein‐coding genes with RNA polymerase II (Pol II) and contain exons and/or introns from parental genes (Kristensen et al. 2019). They are dynamically expressed in a specific manner (Rybak‐Wolf et al. 2015) and control a variety of biological functions, such as cell proliferation, apoptosis, differentiation, and metabolism (Chen 2020).

CircRNAs have progressively shown the ability to alter the expression and function of target genes, which are implicated in several diseases, such as neurological disorders, cancer, metabolic disorders, cardiovascular disorders, and pregnancy‐related diseases (Gao et al. 2021). CircRNAs have been identified within ovaries (Zhang et al. 2022) and have been shown to exhibit expression patterns relevant to developmental stages during follicular development (Shen et al. 2020), which raises questions about their involvement in ovarian hyperthecosis. Through a variety of mechanisms, circRNAs have been shown to play a crucial regulatory function in the development of ovarian hyperthecosis (Chen et al. 2022). CircRNAs may therefore become useful therapeutic targets and precise diagnostic markers for ovarian hyperthecosis. However, there is currently insufficient data to determine the precise association between circRNAs and ovarian hyperthecosis, and it is unclear how circRNAs might be used to diagnose and treat ovarian hyperthecosis.

Medicinal plants are increasingly recognized for their therapeutic potential in the treatment of ovarian hyperthecosis (Akter et al. 2023). *Amaranthus spinosus* Linn., commonly referred to as “pigweed” and belonging to the Amaranthaceae family, exhibits a diverse array of medicinal properties. This plant has been utilized in the management of various conditions, including inflammatory disorders, malaria, bacterial infections, diuretic needs, viral diseases, and hepatic ailments. Numerous studies have been conducted on *A. spinosus* , exploring its extensive pharmacological effects, which encompass antinephritic, antidiabetic, antitumor, analgesic, antimicrobial, anti‐inflammatory, spasmolytic, bronchodilator, hepatoprotective, spermatogenic, antifertility, antimalarial, and antioxidant activities, among others (Jhade et al. 2009). Phytochemical analysis revealed the presence of several active compounds, such as alkaloids, flavonoids, phenolic acids, steroids, amino acids, terpenoids, lipids, saponins, tannins, and carotenoids (Akinloye et al. 2023).

Metabolomics, which focuses on identifying and quantifying all metabolites in biological systems, provides a powerful tool for discovering biomarkers to diagnose ovarian hyperthecosis and investigate changes in biochemical pathways. Unlike traditional biochemical methods that examine only a few markers at once, metabolomics allows for the collection of quantitative data on a wide range of metabolites, offering a comprehensive perspective on metabolism and metabolic changes linked to disease. By analyzing differences in metabolite levels between disease and healthy controls, metabolomics can identify metabolites associated with diseases. These altered metabolites could serve as diagnostic biomarkers, enabling early detection and preventive approaches (Song et al. 2021).

Given the reported antioxidant and anti‐inflammatory properties of *A. spinosus* , the main aim of this study was to assess the effect of its EtOH extract on Dehydroepiandrosterone (DHEA) induced ovarian hyperthecosis in a rat model. This study presents a multiplex approach to the relationships between circRNAs and metabolomics towards a better understanding of ovarian hyperthecosis management and diagnosis based on differential biomarkers involved in the treatment.

## 2 Materials and Methods

### 2.1 Plant Collection, Authentication and Preparation

In the flowering season of April 2022, *A. spinosus* whole plant was harvested from the El Menoufia governorate (30°20′49″ N 30°52′14″ E) near the Nile delta of Egypt. The botanical sample was authenticated by Prof. Ahmed M. Abdel Gawad, a professor of plant ecology, and deposited into the Faculty of Science Herbarium at Mansoura University in Egypt bearing voucher code AMSPxZG‐677‐RxY/22‐06652.

### 2.2 Extraction and Chemical Analysis Procedures

After being cleaned and allowed to air dry for 2 weeks at 25°C and 60% relative humidity, all of the collected quantities were crushed into a fine powder using an electric grinder. The 750 g of air‐dried plant material was macerated for a week with 4 L of a mixture of ethanol‐distilled water (7:3) and filtered. The overall extract was pooled together and vacuum‐dried, affording 25.9 g of black gum that was kept at 4°C till further analysis. *A. spinosus* EtOH extract was subjected to metabolites characterization utilizing ultra‐performance liquid chromatography‐mass spectrometry analysis (UHPLC‐ESI‐Orbital Trap‐MS) following the previously established methodology (Taher et al. 2025).

### 2.3 Chemical and Materials

Dehydroepiandrosterone (DHEA) was obtained from Sigma‐Aldrich Co (St. Louis, MO, USA). LH and testosterone ELISA kits were purchased from (R&D Systems, USA).

### 2.4 Animals and Treatment

Postpubertal Western female Albino rats 42‐day‐old (No. 32) weighing 100–120 g from the National Research Center's animal house were utilized in the present investigation. The animals will be raised under controlled circumstances (22 5°C, 55% humidity, and a 12‐h light/dark cycle). Animals were provided with access to water and a typical diet. All animal care and treatment techniques closely follow the ethical protocols and policies established by the Animal Care and Use Committee of the National Research Center and the US National Institutes of Health, as per the approval number 04481223.

### 2.5 Experimental Design

Proceeding acclimatization, animals were divided into four cages (eight rats). Grouping and dosing details are given in Table 1.

**TABLE 1:** Experimental design including disease induction and treatment phase.

Groups	Duration (8 weeks)
*Disease induction phase*	
G1: Control group	Olive oil was administered orally
G2: DHEA	6 mg/100 g body weight of DHEA was dissolved in olive oil, injected subcutaneously and administered for 1 month along induction and treatment period (Kim et al. 2018)
*Treatment phase, duration (5th–8th week)* Post DHEA induction the following treatment strategy was applied as described below	
G3: *A. spinosus* low dose	50 mg/kg BW was administered orally
G4: *A. spinosus* high dose	100 mg/kg BW was administered orally

### 2.6 Blood Sampling and Tissue Preparation

Rats were weighed and sedated (with 75% carbon dioxide). After gathering sera from the retro‐orbital vein, samples were centrifuged at 5000 rpm for 10 min before being preserved at −80°C. Rats were euthanized, and ovarian tissue was isolated and kept in 10% formaldehyde for histopathological analysis.

### 2.7 Measured Biochemical Parameters

#### 2.7.1 Serum Glutathione and Malondialdehyde

Oxidative stress biomarkers, serum glutathione (GSH) and malondialdehyde (MDA) assay kits were provided from the Randox Company, assessed by measuring the produced colored product spectrophotometrically (Jasco V‐730 Spectrophotometer) at 450 and 505 nm, respectively, by the method of Bakan et al. (2003).

#### 2.7.2 ELISA Assay of Serum LH, FSH and Testosterone

Using an enzyme‐linked immunosorbent assay kit (R&D Systems, MN, USA) in accordance with the manufacturer's instructions, the activities of LH and testosterone were determined. After that, a quantitative sandwich enzyme immunoassay was used to assess the experiment. The plate was precoated with the suitable antibodies. Subsequently, the LH, FSH, and testosterone‐specific enzyme‐linked secondary antibody was added, which was followed by the immobilized antibody. At 450 nm, the absorbance was then determined. The Agilent BioTek Microplate reader, Neo2, was used to measure the color intensity at 450 nm (Kadry and Abdel Megeed 2022).

### 2.8 Circular—RNA (hsa‐circ‐0001577) Analysis

Circ‐RNA was extracted utilizing a nucleic acid extraction kit (NucleoSpin REF. 740901.250) purchased from Macherey‐Nagel GmbH & Co. KG, Germany. Serum lysis occurs via an equal volume of RA1 buffer and β‐mercaptoethanol. Reducing the viscosity and clearing the lysate, filtration was done using NucleoSpin Filter. A violet ring was placed in the collection tube, then 70% ethanol was added to the homogenized lysate. Further, the NucleoSpin RNA Column (light blue ring) was placed in a collection tube, then the lysate was loaded into the column and centrifuged. Three hundred and fifty microliter of membrane desalting buffer (MDB) was added and centrifuged to dry the membrane; then further RA3 buffer was added. The NucleoSpin RNA Column was placed into a nuclease‐free collection tube and RNA was eluted in 60 μL RNase‐free H_2_O. The purity (A260/A280 ratio) and the concentration of Circ‐RNA were determined using spectrophotometry (dual wave length Beckman, Spectrophotometer, USA). Then Quantitative real‐time polymerase chain reaction (qRT‐PCR) was assessed using kits provided by Bioline, a median life science company, UK (SensiFAST SYBR Hi‐ROX One‐Step Kit, catalog no. PI‐50217 V): Primer sequences for the studied target gene hsa_circ_0001577 and reference housekeeping gene (GAPDH) were shown in Table 2 (Kadry and Abdel Megeed 2023).

**TABLE 2:** Primer's sequence of all studied genes.

Gene symbol	Primer sequence from 5′ to 3′
hsa_circ_0001577	F: GGGCTTCAAACACCAGGAGA R: GTGCTTCCTTTGCCTGATGC NM_005493.3
GAPDH	F: GAAAGCCTGCCGGTGACTAA R: GCGCCCAATACGACCAAATC NM_001256799.3

### 2.9 Serum Metabolomics Analysis

#### 2.9.1 Sample Preparation

A volume of 200 μL of acetonitrile was introduced into serum samples measuring 100 μL. Subsequently, the mixture was subjected to centrifugation. Then, the supernatant obtained from the experiment was dried under vacuum. The dried pellet was incubated for 1 h at 60°C with 20 mg/mL of a 50 μL methoxyamine HCl/pyridine mixture, and the metabolites were derivatized by adding 100 μL of MSTFA containing 1% TMS to the mixture and incubating for 1 h at 60°C. To maintain consistency in testing, quality control samples (QC) were conducted from each sample. This QC sample was then utilized to evaluate the repeatability and stability of the analytical platform. Gas chromatography was employed for metabolites profiling (Thermo Scientific Corp., USA) connected to a mass spectrometer as a detector (ISQ Single Quadrupole Mass Spec‐trometer). The method described by Ammar et al. (2022) was used to perform the chromatographic separation. By mass matching to the NIST library database and comparing the retention indices (RI) of the serum metabolites to standards for n‐alkanes (C7–C40), the serum metabolites were identified. The AMDIS software (www.amdis.net) de‐convoluted the peaks before mass spectral matching. Before conducting multivariate data analysis, the abundance of mass signals was normalized, subjected to Pareto scaling, and then examined using SIMCA software performing PCA (principal component analysis) and OPLS‐DA (orthogonal projection to latent structure discriminate analysis).

### 2.10 Histopathological Analysis

Ovary tissues were fixed in 10% formaldehyde and embedded in paraffin. Sections of 5 mm thickness were stained with hematoxylin and eosin (H&E), and then were examined under a light microscope for the determination of pathological changes (Suvarna et al. 2018).

#### 2.10.1 Histopathological Lesion Scoring

Histopathological alterations of ovaries were recorded and scored as no changes (0), mild (1), moderate (2) and severe (3) changes; the grading was determined by percentage as follows: < 30% changes (mild change), < 30%–50% (moderate change), and > 50% (severe change) (Korany et al. 2019).

#### 2.10.2 Statistical Analysis

The results of biochemical analysis were analyzed using computer program Statistical Package for the Social Sciences (SPSS, Chicago, IL, USA) software version 20. Data were analyzed using one‐way ANOVA followed by Tukey's post hoc test; the significance between means of the studied groups were values of less than 0.05 regarded as statistically significant. Quantitative values were expressed by mean ± SEM. Multivariate data analysis was performed using SIMCA‐P 14.1 software (Umetrics, Umea, Sweden). Orthogonal partial least squares‐discriminant analysis (OPLS‐DA). The OPLS‐DA method helps visualize distinguishing metabolites and aids in classifying test samples (Farag et al. 2021). The variable importance in projection (VIP) is used to evaluate the contribution of each variable to the OPLS‐DA model. Variables with a VIP value above 1.0 are regarded as significant in differentiating between the groups under study. It is reported that significant coefficient *p* values < 0.05 and fold‐changes (FC) > 2.0 (or < 0.5) were selected after applying univariate analysis of *t*‐test and fold‐change to the metabolites using Metaboanalyst 6.0 platform (https://www.metaboanalyst.ca/) (Farag et al. 2022). It was also utilized to conduct pathway analysis by exporting the key differential metabolites.

## 3 Results

### 3.1 Metabolites Profiling

Secondary metabolites of *A. spinosa* EtOH extract were determined using UPLC‐MS analysis (Figure S1). The chromatographic separation led to the identification of 72 metabolites in negative mode listed, and their mass data are described in Table 3. The detected metabolites belong to four major classes, namely fatty acids, organic acids, phenolic acids and their derivatives, as well as amino acids, with 28, 12, 11, and 8 peaks detected respectively. Followed by other minor classes, including triterpenoidal saponins, flavonoids, and pyridines.

**TABLE 3:** Metabolites identified in *A. spinosa* using UPLC/PDA/ESI‐qTOF‐MS in negative ionization modes.

Peak no.	RT	[M‐H]^−^	Error (ppm)	Compound name	MS/MS	Class
1	0.91	195.0513	−1.34	Gluconic acid	177.04, 129.01, 87.01, 75.01	Organic acid
2	0.93	165.0405	−0.5	Ribonic acid	147.03, 129.01	Organic acid
3	0.993	209.0668	−0.64	Mannoheptulose	181.06, 159.02, 123.01	Sugar
4	1.019	115.0037	−0.43	Maleic acid	101.06, 73.02	Organic acid
5	1.051	191.0199	−0.26	(*iso*)Citric acid	173.01, 147.02, 111.01	Organic acid
6	1.058	133.0146	−2.64	Malic acid	115.01, 107.03, 71.01	Organic acid
7	1.069	205.0356	−0.96	Homocitric acid	193.03, 179.01, 129.01, 111.01	Organic acid
8	1.071	105.0194	−0.13	Dihydroxypropanoic acid	75.01	Organic acid
9	1.155	117.0195	−0.93	Succinic acid	73.03	Organic acid
10	1.565	186.041	−1.09	Acetyl L‐glutamate	143.05	Amino acid
11	1.587	128.0355	−1.42	Pyroglutamic acid	82.02	Amino acid
12	3.44	131.0351	−0.75	Glutaric acid	115.01	Organic acid
13	4.559	164.0719	−1.2	Phenylalanine	103.05	Amino acid
14	6.171	153.0193	0.8	Protocatechuic acid	109.02	Phenolic acid
15	7.809	144.0667	−0.57	3‐Hydroxyquinoline	83.05	Quinoline
16	8.048	329.0879	−0.12	Vanilloyl glucose	167.03	Phenolic
17	8.384	151.0402	1.85	Vanillin	136.04, 108.02, 71.01	Phenolic aldehyde
18	8.512	137.0246	−1.17	Monohydroxybenzoic acid	115.01, 93.03	Phenolic acid
19	8.629	323.126	8.84	Glabranin	305.11, 283.11, 255,06, 163.04	Flavonoid
20	8.808	175.0614	−1.08	Isopropylmalic acid	133.04, 115.04, 85.05	Organic acid
21	8.914	182.0461	−1.19	Unknown	138.05, 123.03, 71.02	Unknown
22	9.036	172.0981	−1.06	Acetylleucine	159.08, 129.08, 113.06, 69.03	Amino acid
23	9.071	315.1085	0.67	Vanilloloside	244.06, 177.12, 159.11	Phenolic
24	9.242	159.0664	0.12	Heptanedioic acid	115.07, 97.06	Fatty acid
25	9.507	158.0823	−0.21	*N*‐Isovalerylglycine	136.07, 65.01	Amino acid
26	9.602	236.0561	1.46	*N*‐Benzoyl‐DL‐aspartic acid	221.07, 177.06, 164.07, 147.04, 131.05	Amino acid
27	9.643	250.0722	−0.41	*N*‐Feruloylglycine	189.05, 166.01, 132.03, 115.01, 88.04	Amino acid
28	9.784	297.0651	−10.45	Benzoylglucuronide	253.11, 217.11, 185.04, 155.11, 111.08	Phenolic
29	10.023	206.0824	−0.64	*N*‐Acetyl‐L‐phenylalanine	163.07, 147.04, 103.05	Amino acid
30	10.094	303.0909	−10.24	Methyl catechin	263.13, 225.15, 201.11, 165.01, 151.01, 129.05	Flavonoid
31	10.112	173.0821	−1.17	Suberic acid	129.09, 111.08	Fatty acid
32	10.144	167.0351	−0.51	Vanillic acid	153.03, 123.03, 109.03	Phenolic acid
33	10.201	195.0638	12.61	Acetosyringone	177.05, 151.07, 127.07	Phenolic
34	10.318	161.0246	−0.91	3‐Hydroxycoumarin	133.03, 117.03, 77.03	Coumarin
35	10.409	413.146	−1.35	Unknown	269.11, 161.04, 125.02	Unknown
36	10.415	217.1083	−0.67	Hydroxydecanedioic acid	199.09, 171.11	Fatty acid
37	10.511	271.1914	0.5	Pentadecanedioic acid	237.11, 219.11, 183.13, 171.11, 127.07	Fatty acid
38	10.53	337.0929	0.26	5‐p‐Coumaroylquinic acid	205.05, 191.05, 173.05, 145.02, 129.01	Phenolic
39	10.648	237.0406	−0.61	Benzoyl malic	177.10, 121.02, 77.04	Phenolic
40	10.652	121.0296	−1.06	Benzoic acid	103.05, 77.04	Phenolic acid
41	10.835	359.207	1.55	Unknown	305.17, 225.11, 183.11, 159.06, 111.11	Unknown
42	10.928	463.2547	0.56	Kaempferol glycoside	285.05	Flavonoid
43	10.978	187.0979	−1.55	Azelaic acid	125.09	Fatty acid
44	11.009	209.0798	10.38	Sinapyl alcohol	165.08, 141.09	Alcohol
45	11.043	243.124	−0.14	Oxododecanedioic acid	225.11, 201.11, 181.12	Fatty acid
46	11.173	145.0871	−0.73	Hydroxyheptanoic acid	127.07	Fatty acid
47	11.199	138.0197	−0.24	Hydroxypicolinic acid	123.03, 94.03	Nitrogenous compounds
48	11.256	273.1707	−0.07	Hydroxytetradecanedioic acid	255.16, 211.17, 187.05	Fatty acid
49	11.518	199.0976	0.64	Decenedioic acid	181.17, 155.11, 137.09	Fatty acid
50	11.727	159.1026	0.74	Hydroxyoctanoic acid	115.07, 97.06	Fatty acid
51	11.768	201.1134	−0.69	Decanedioic acid (sebacic acid)	183.11, 139.11	Fatty acid
52	11.968	231.1239	−0.26	Hydroxyundecanedioic acid	213.09, 187.23	Fatty acid
53	12.041	301.2025	−1.51	Hydroxyhexadecanedioic acid	257.11, 143.07	Fatty acid
54	12.351	245.1393	1.18	Hydroxydodecanedioic acid	227.23, 183.27	Fatty acid
55	12.503	227.1291	−0.95	Dodecenedioic acid	209.11, 183.14, 165.12	Fatty acid
56	12.553	215.1289	−0.39	Undecanedioic acid	199.12, 173.09	Fatty acid
57	12.663	345.2282	0.19	Dihydroxyoctadecanedioic acid	327.21, 309.21	Fatty acid
58	12.685	327.2177	0.07	Trihydroxyoctadecadienoic acid	309.21, 283.25, 265.18	Fatty acid
59	12.872	331.2488	0.61	Trihydroxyoctadecanoic acid	313.23, 287.14	Fatty acid
60	13.028	329.2336	−0.81	Pinellic acid	293.21, 265.21, 229.14, 211.12, 171.11, 139.11	Fatty acid
61	13.202	661.3227	0.43	Celosin F	617.37, 501.32, 439.32, 393.18, 347.18, 255.41, 193.03	Saponin
62	13.221	663.3747	0.38	Medicagenic acid‐3‐*O*‐glucopyranoside	599.28, 543.32, 501.32, 439.32, 193.03	Saponin
63	13.603	309.2071	−1.33	Porrigenic acid	291.19, 221.15, 183.11	Fatty acid
64	13.74	675.3382	0.63	Celosin E derivative	631.34, 501.32, 455.32, 407.18, 193.03	Saponin
65	13.896	677.3541	0.51	Celosin E	577.03, 519.25, 501.32, 319.49, 193.03	Saponin
66	14.759	311.2228	0.14	Octadecenedioic acid	293.21, 267.14, 249.22	Fatty acid
67	15.505	313.2385	−0.32	Octadecanedioic acid	295.22, 277.21, 269.21	Fatty acid
68	16.262	293.2122	−0.17	Hydroxylinolenic acid	275.21, 249.22, 231.22	Fatty acid
69	16.915	295.2278	−0.15	Hydroxy‐octadecadienoic acid	277.21, 233.22	Fatty acid
70	17.597	297.2436	−0.18	Ricinoleic acid	279.23	Fatty acid
71	17.955	269.2124	−0.7	Oxohexadecanoic acid	251.19, 225.22, 207.27	Fatty acid
72	20.15	313.2751	−0.65	Hydroxynonadecanoic acid	295.26, 269.27, 251.23	Fatty acid

#### 3.1.1 Fatty Acids

Fatty acids represented the most abundant class represented by 28 peaks. Their abundance can be observed in the total ion chromatogram within the elution range of *t*_R_: 11–20 min. This late elution is consistent with their nature as nonpolar compounds compared to other metabolite classes. All fatty acids were identified with their parent molecular formula and product ions yielded after showing a loss of a water molecule (−18 amu), a loss of carboxylic moiety (−44 amu), or a loss of both (−62 amu), to include suberic and azelaic acids detected in peaks 31 and 43 with [M‐H]^−^ at *m*/*z* 173.0821 and 187.0979 and a molecular formula of (C_8_H_14_O_4_) and (C_9_H_16_O_4_) respectively. Similarly, pinellic and porrigenic acids were detected in peaks 60 and 63 with [M‐H]^−^ at *m*/*z* 329.2336 and 309.2071 and a molecular formula of (C_18_H_34_O_5_) and (C_18_H_30_O_4_) respectively. Hydroxylinolenic acid was detected in peak 68 with [M‐H]^−^ at *m*/*z* 293.2122 (C_18_H_30_O_3_) with product ions at *m*/*z* 275.21 [M‐H_2_O‐H]^−^, *m*/*z* 249.22 [M‐COO‐H]^−^, and *m*/*z* 231.22 [M‐H_2_O‐COO‐H]^−^. Among detected fatty acids, octadecanedioic acid was annotated in peak 67 with [M‐H]^−^ at *m*/*z* 313.2385 and product ions at *m*/*z* 295.22 [M‐H_2_O‐H]^−^, *m*/*z* 277.21 [M‐COO‐H]^−^, and *m*/*z* 269.21 [M‐H_2_O‐COO‐H]^−^. A similar fragmentation pattern was observed in peak 57 assigned as its dihydroxy derivative. Other detected fatty acids are described along with their mass spectral data in Table 3.

#### 3.1.2 Phenolics and Organic Acids

Represented by 22 peaks, phenolics and organic acids were the second most abundant classes observed in *A. spinosa* . Their abundance is visible within the elution region of *t*_R_: 1–8 min (Figure S1) consistent with being the most polar among detected metabolites. Peak 5 showed a molecular ion [M‐H]^−^ at *m*/*z* 191.0199 (C_6_H_8_O_7_) with product ions at *m*/*z* 173.01 [M‐H_2_O‐H]^−^, *m*/*z* 147.02 [M‐COO‐H]^−^, and *m*/*z* 111.01 [M‐2H_2_O‐COO‐H]^−^, and was identified as (iso)citric acid. A similar fragmentation pattern was observed in peak 7 and was assigned as homocitric acid. Likewise, malic acid was detected in peak 6 with [M‐H]^−^ at *m*/*z* 133.0146 (C_4_H_6_O_5_) and a product ion at *m*/*z* 115.01 [M‐H_2_O‐H]^−^, and *m*/*z* 71.01 [M‐H_2_O‐COO‐H]^−^. Similarly, peak number 9 showed a molecular ion at *m*/*z* 117.0195 (C_4_H_6_O_4_) with a product ion at *m*/*z* 73.01 [M‐COO‐H]^−^, and was annotated as succinic acid. Vanillic acid and its reduced form vanillin were among the detected phenolics in peaks 32 and 17 with [M‐H]^−^ at *m*/*z* 167.0351 and 151.0402, respectively. Both showed a characteristic product ion at *m*/*z* 123.03 which corresponds to the loss of their functional groups, namely carboxylic [M‐COO‐H]^−^ and aldehydic [M‐CO‐H]^−^ moieties for vanillic acid and vanillin respectively. Similar fragmentation patterns were observed with peaks 16 and 23, which were assigned as their derivatives. Another phenolic acid was detected in peak 14 with [M‐H]^−^ at *m*/*z* 153.0193, C_7_H_6_O_4_ and a product ion at *m*/*z* 109.02 [M‐COO‐H]^−^ annotated as protocatechuic acid.

#### 3.1.3 Amino Acids and Nitrogenous Compounds

As depicted from UPLC‐MS analysis, 10 amino acids, their derivatives, as well as pyridines were detected, including peak 11 with [M‐H]^−^ at *m*/*z* 128.0355 (C_5_H_7_NO_3_) with a product ion at *m*/*z* 82.02 [M‐HCOO‐H]^−^, annotated as pyroglutamic acid. Peak 13 showed a molecular ion [M‐H]^−^ at *m*/*z* 164.0719 (C_9_H_11_NO_2_) with a product ion at *m*/*z* 103.05 [M‐COO‐NH_2_‐H]^−^, and was annotated as phenylalanine. Three acetyl amino acid derivatives were detected in peaks 10, 22, and 29, showing a loss of acetyl moiety (−43 amu) and were annotated as acetyl L‐glutamate, acetylleucine, and *N*‐acetyl‐L‐phenylalanine, respectively. Aside from amino acids, pyridines represented another class of nitrogenous metabolites identified in *A. spinosa* ; in particular, peak 21 showed a molecular ion [M‐H]^−^ at *m*/*z* 182.0461 (C_8_H_9_NO_4_) with product ions at *m*/*z* 138.05 [M‐COO‐H]^−^ and *m*/*z* 123.03 [M‐COO‐CH_3_‐H]^−^, and was identified as pyridoxic acid. Also, hydroxy picolinic acid was identified in peak 47 with [M‐H]^−^ at *m*/*z* 138.0197 (C_6_H_5_NO_3_) with a product ion at *m*/*z* 94.03 [M‐COO‐H]^−^.

#### 3.1.4 Triterpenoid Saponins

Triterpenoid saponins are known to be present abundantly within species belonging to the family Amaranthaceae. Many reported biological activities of Amaranthaceae members have been attributed to the presence of such class (Zehring et al. 2015). Structural elucidation of saponins was confirmed by characteristic product ions yielded upon the fission of the saponin aglycone and sugar moieties. For example, medicagenic acid‐3‐*O*‐glucopyranoside (C_36_H_56_O_11_) and celosin E (C_36_H_54_O_12_) were detected in peaks 62 and 65 with [M‐H]^−^ at *m*/*z* 663.3747 and 677.3541, and a characteristic product ion at *m*/*z* 501.32 corresponding to the saponin aglycone after the loss of hexose (−162 amu) and glucuronate (−176 amu) respectively.

### 3.2 Bioassay Results

#### 3.2.1 circRNA (hsa‐circ‐0001577) Modulation

**FIGURE 1:**
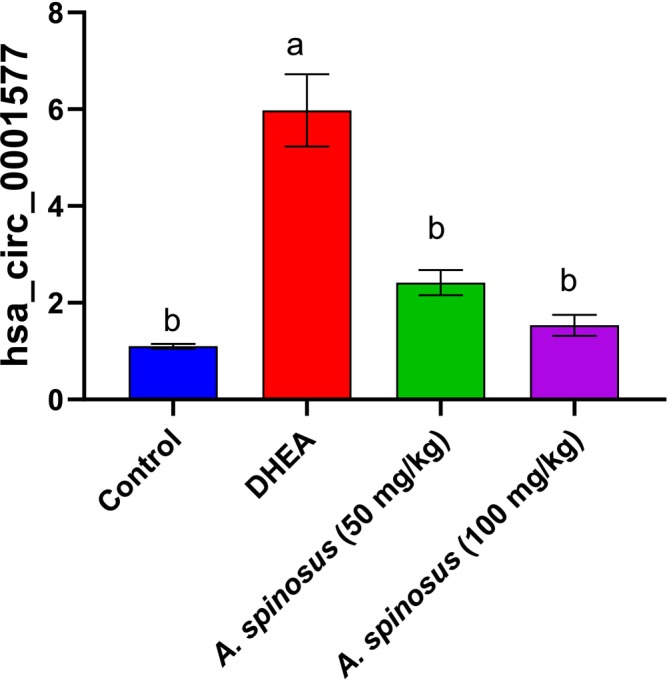
Effect of treatment with *Amaranthus spinosus* EtOH extract (50 and 100 mg/kg, orally, once daily, for 4 weeks) on circRNA (hsa‐circ‐0001577) level. Values are expressed as mean ± SEM, *n* = 6. Data were analyzed using one‐way ANOVA followed by Tukey's post hoc test, with different letters indicating significant differences at *p* < 0.05).

As shown in Figure 1, ovarian hyperthecosis induced via DHEA revealed a significant upregulation in circRNA (hsa‐circ‐0001577) with a fold change of (5.4). In contrast, treatment with *A. spinosus* at low and high doses downregulated circRNA (hsa‐circ‐0001577) with the high dose superiority with a fold change (1.4).

#### 3.2.2 Modulation of Oxidative Stress Biomarkers

**FIGURE 2:**
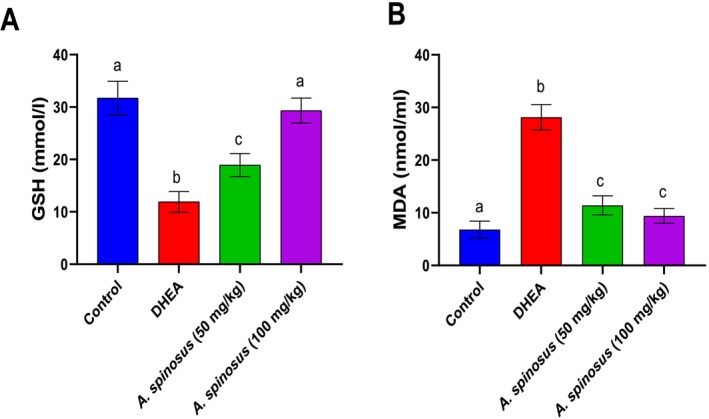
Effect of *Amaranthus spinosus* EtOH extract (50 and 100 mg/kg, orally, once daily, for 4 weeks) on GSH level (A) and MDA activity (B). Values are expressed as mean ± SE, *n* = 6. Data were analyzed using one‐way ANOVA followed by Tukey's post hoc test, with different letters indicating significant differences at *p* < 0.05.

Oxidative stress biomarkers including GSH and MDA were assayed and revealing significant GSH reduction with a mean value of (11.9 ± 1.99), concurrent with MDA elevation with a mean value of (28.14 ± 2.4) post DHEA intoxication. In contrast, *A. spinosus* at low and high doses modulated these parameters, with the high dose superiority at (29.3 ± 2.4 for GSH and 9.4 ± 1.4 for MDA) (Figure 2).

#### 3.2.3 Modulation of Hormonal Imbalance

**FIGURE 3:**
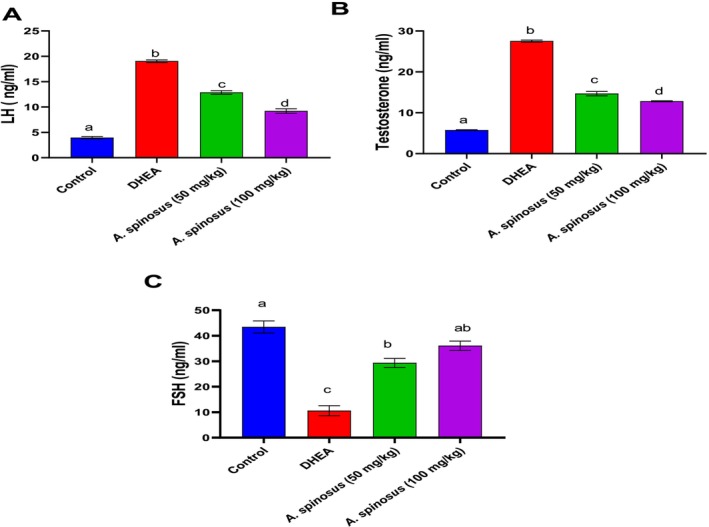
Effect of *Amaranthus spinosus* EtOH extract (50 and 100 mg/kg, orally, once daily, for 4 weeks) on LH (A), testosterone (B), and FSH (C) levels. Values are expressed as mean ± SE, *n* = 6. Data were analyzed using one‐way ANOVA followed by Tukey's post hoc test, with different letters indicating significant differences at *p* < 0.05.

Hormonal biomarkers including luteinizing hormone and testosterone revealed a significant elevation with a mean value of (19 ± 0.25 and 27.54 ± 0.24) respectively, concurrent with FSH reduction with a mean value of (10.6 ± 1.96) post DHEA intoxication. Treatment with *A. spinosus* at both doses modulated this hormonal disturbance with the high dose superiority at 9.198 ± 0.45 for LH, 12.80 ± 0.12 for testosterone, and 36.06 ± 1.80 for FSH (Figure 3).

#### 3.2.4 Histopathological Assay and Lesion Score

**FIGURE 4:**
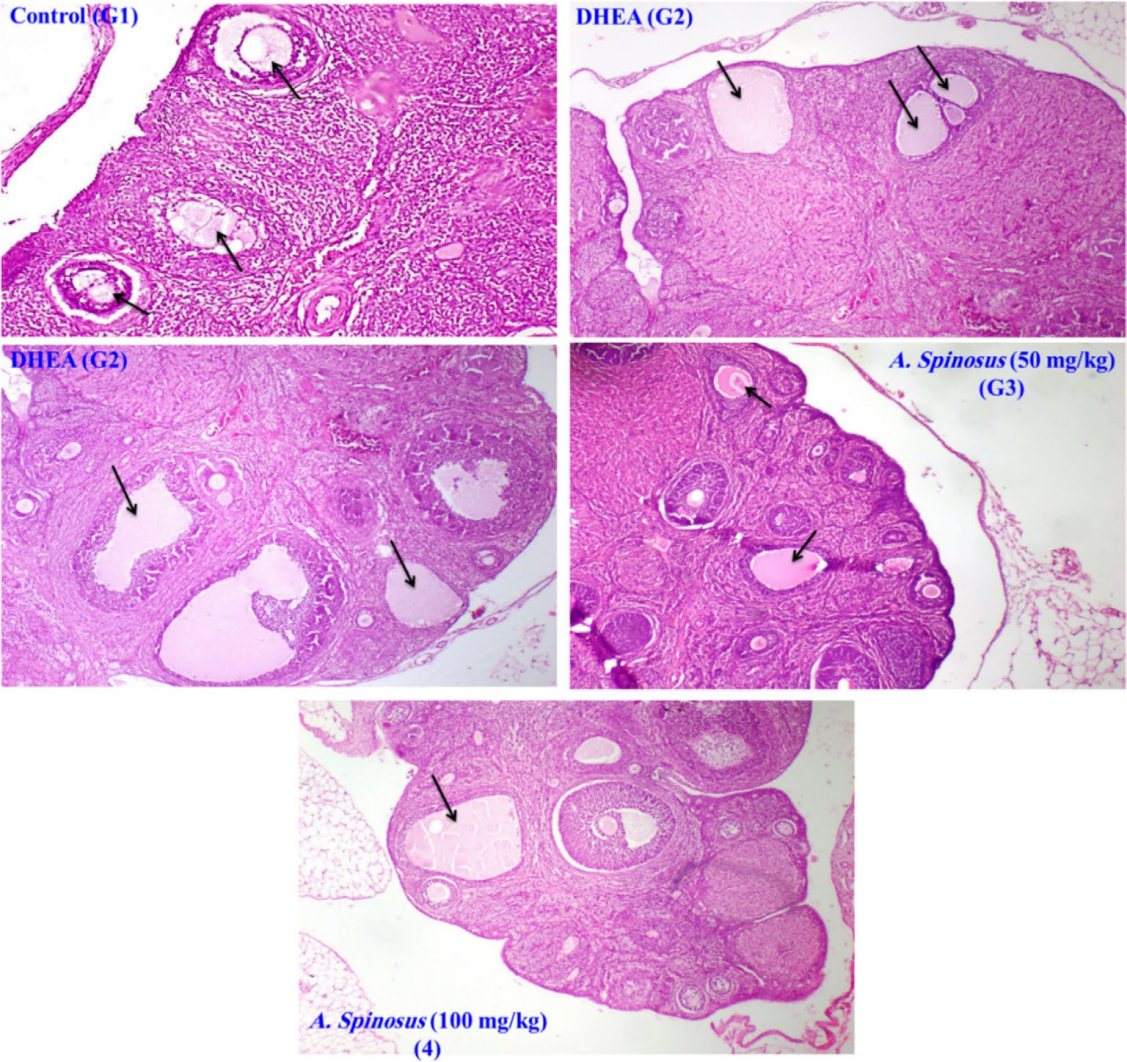
Photomicrographs of: Control untreated group (G1): Rat ovary showing normal histological structure of ovarian follicles (arrows), DHEA group (G2): Rat ovary showing multiple ovarian follicular cysts (arrows), DHEA group (G2): Rat ovary showing multiple follicular cysts (arrows), *Amaranthus spinosus* EtOH (50 mg/kg) group (G3): Rat ovary showing follicular cysts (arrows), and *A. spinosus* EtOH (100 mg/kg) group (G4): Rat ovary showing only one follicular cyst (arrows); (H&E ×100).

To confirm biochemical assay results for the ameliorative effect of *A. spinosus* , histopathological examination of the ovary and kidney was attempted. Group 1 showed a normal histological structure of ovarian follicles. In contrast, group 2 revealed the presence of multiple ovarian cysts (polycystic ovaries). Group 3 showed mild improvement in the form of fewer follicular cysts with moderate vacuolar degeneration. Group 4 revealed noticeable amelioration as the ovarian cysts were very few (Figure 4). Recorded lesions in both ovaries were scored according to their severity (Table 4).

**TABLE 4:** Scoring of histopathological alterations in ovaries of all treated groups.

Lesions	Groups			
	G1	G2	G3	G4
Ovarian cysts	0	3	2	1

*Note:* The score system was designed as: score 0 = absence of the lesion in all rats of the group (*n* = 5), score 1 = (< 30%), score 2 = (< 30%–50%), score 3 = (> 50%). Control untreated group (G1); DHEA group (G2); *A. spinosus* EtOH (50 mg/kg) group (G3); *A. spinosus* EtOH (100 mg/kg) group (G4).

### 3.3 Serum Metabolomics for Unraveling Metabolic Variations Between Ovarian Hyperthecosis‐Diseased and *A. spinosus* ‐Treated Rats

**FIGURE 5:**
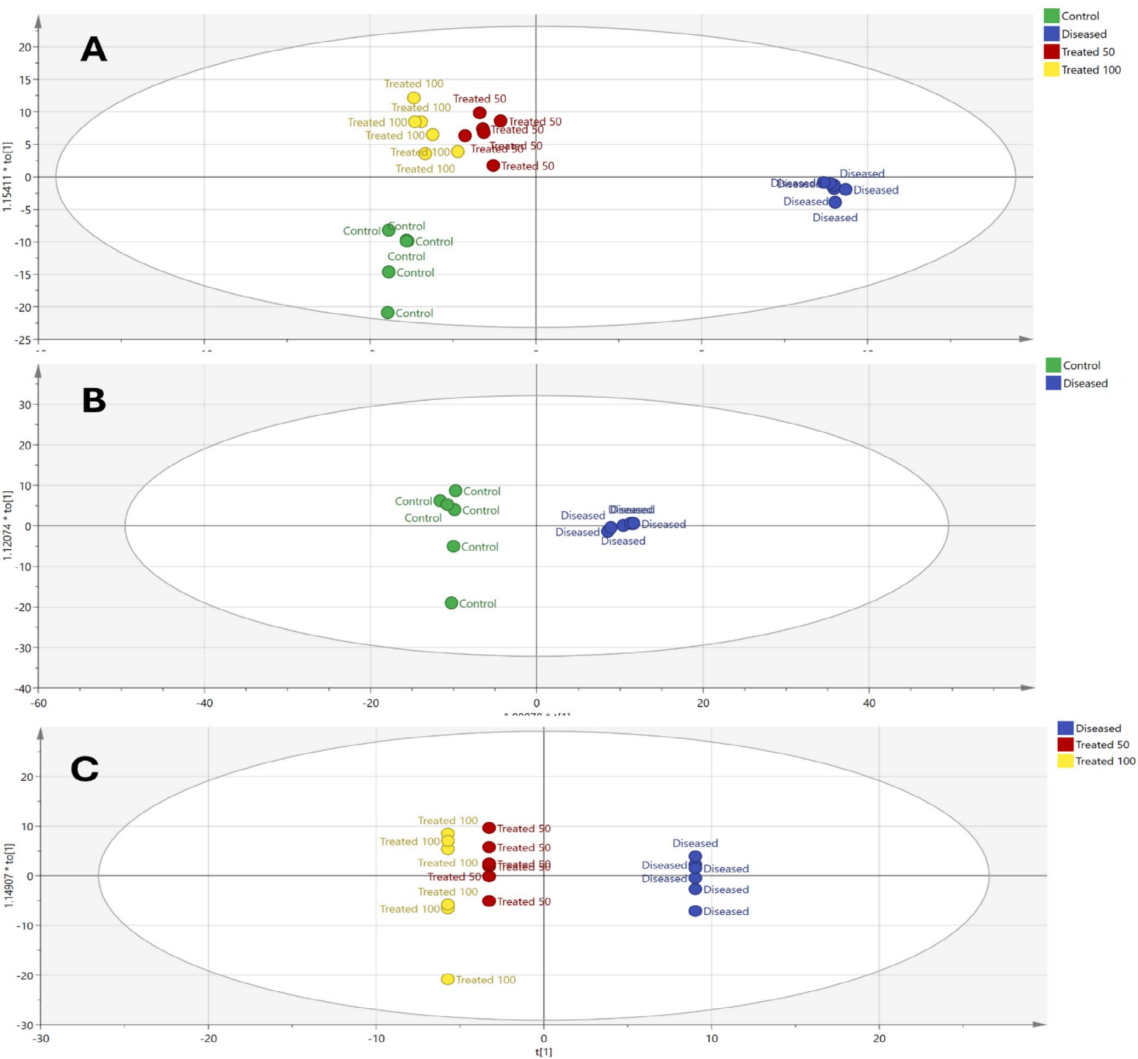
Score scatter plot of OPLS‐DA models (A) all four groups (control, ovarian hyperthecosis‐diseased, and dose‐based *Amaranthus spinosus* ‐treated groups), (B) control and ovarian hyperthecosis‐diseased groups, and (C) ovarian hyperthecosis‐diseased and dose‐based *A. spinosus* ‐treated groups.

To obtain a more comprehensive information on disease action mechanisms at the metabolite level not previously reported in the literature, GC–MS‐based untargeted serum metabolomics visualized using chemometrics was employed for the first time to assess the mechanisms through which *A. spinosus* might alleviate ovarian hyperthecosis. The supervised orthogonal partial least squares‐discriminant analysis (OPLS‐DA) model of the serum metabolome detected using GC–MS, Figure 5, revealed clear separation between the diseased and control and treated groups, indicating that the ovarian hyperthecosis‐diseased group displays unique metabolic characteristics that can be differentiated from other groups.

**FIGURE 6:**
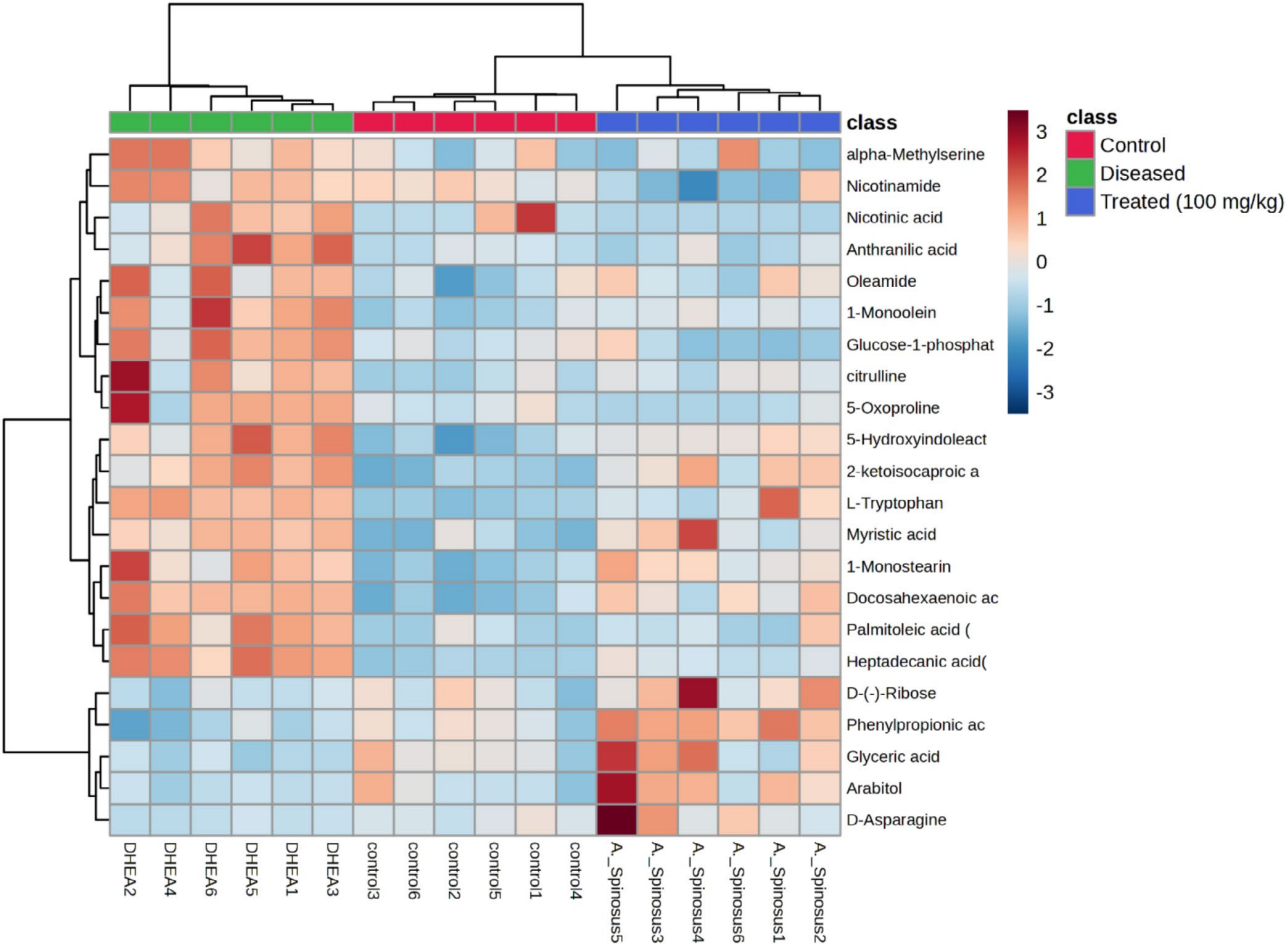
Dendrogram‐heatmap displaying serum metabolic profiles of control, diseased, and after *Amaranthus spinosus* treatment groups.

Further, univariate statistical analysis was implemented to pinpoint the differentially expressed metabolites in the serum of the different studied animal groups. The relative levels of the 22 metabolites, provisionally identified as discriminators among these groups, meeting the criteria of VIP > 1 and a *p* value < 0.05 and fold‐changes (FC) more than 2.0 or less than 0.5 were visually represented in a heatmap following data normalization (Figure 6). By observing the dendrogram, it was evident that the clustering of control and treated groups in one cluster separate from the diseased group indicated the proximity in their chemical composition and restoration of most metabolites to normal level (Figure 6). The list of these key differential metabolites arranged from lowest to highest *p* value, together with their molecular weights, molecular formula, human metabolome database identification number (HMDB ID) and their trends from control (C) to ovarian hyperthecosis‐diseased (D) and from diseased (D) to *A. spinosus* ‐treated (T) groups are detailed in Table S1.

Compared to the healthy control animal group, the ovarian hyperthecosis group showed elevated levels of 17 metabolites, including docosahexaenoic acid, 2‐ketoisocaproic acid, 5‐hydroxyindoleactate, palmitoleic acid (C16:1n7), L‐tryptophan, 1‐monoolein, 1‐monostearin, glucose‐1‐phosphate, anthranilic acid, nicotinamide, 5‐oxoproline, myristic acid, citrulline, oleamide, alpha‐methylserine, and nicotinic acid. In contrast, the diseased (DHEA) group showed significantly lower levels of 5 metabolites, including phenylpropionic acid, arabitol, *D*‐(−)‐ribose, glyceric acid, and *D*‐asparagine.

Interestingly, the *A. spinosus‐*treated demonstrated a notable reduction in the abundance of the 17 elevated metabolites, whereas the 5 reduced metabolites showed a significant increase, resembling levels observed in the healthy control group. These results suggest that *A. spinosus* treatment may help restore the altered metabolites, potentially alleviating the symptoms of ovarian hyperthecosis in rats.

#### 3.3.1 Pathway Enrichment Analysis of Differential Metabolites

**FIGURE 7:**
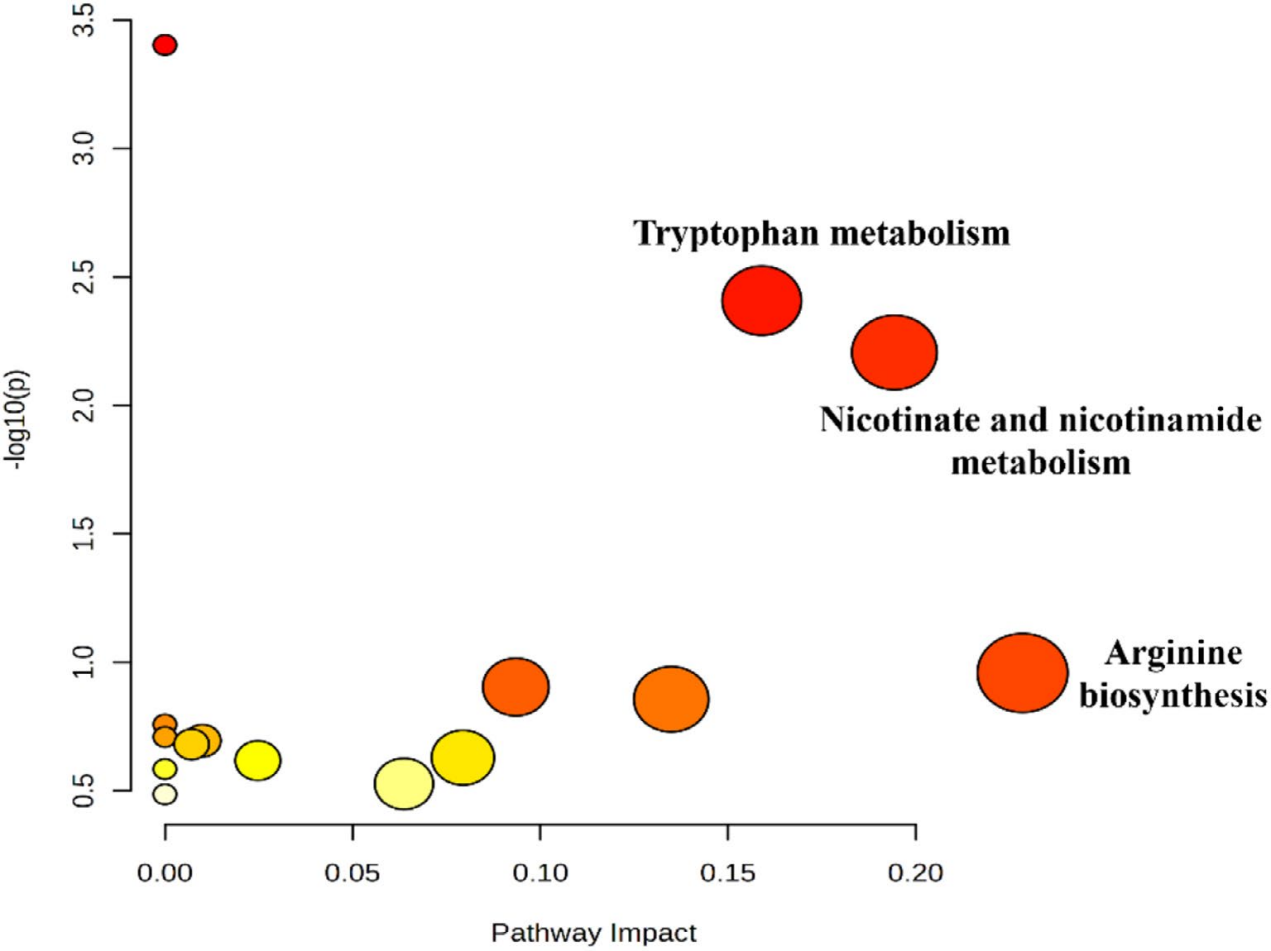
Key metabolic pathways revealed by enrichment analysis of differential metabolites in rats' sera. The node size indicates the pathway impact value.

**FIGURE 8:**
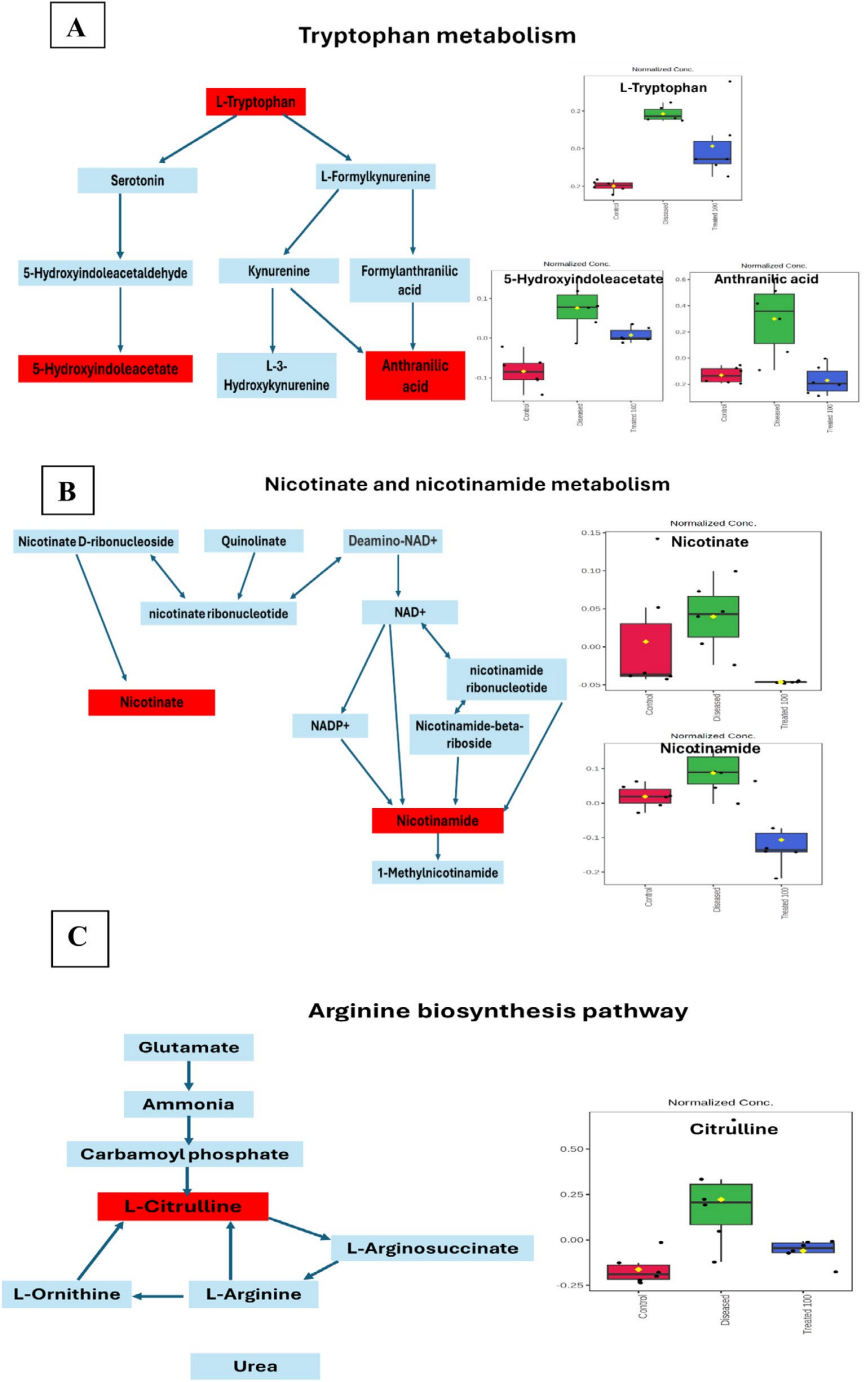
Flow charts illustrating key metabolic pathways (to the left) and box plots (to the right) demonstrating normalized serum levels of differential metabolites in control (red box), diseased (green box), and treated (blue box) groups associated with each enriched pathway: (A) tryptophan metabolism, (B) nicotinate and nicotinamide metabolism, and (C) arginine biosynthesis pathway.

The previously revealed differential biomarkers were further introduced into the MetaboAnalyst 6.0 platform for metabolic pathway enrichment analysis (Figure 7). Based on the enrichment analysis, the significantly affected pathways included tryptophan metabolism, nicotinate and nicotinamide metabolism, and arginine biosynthesis. Box plots of the potential biomarkers involved in those significant pathways were presented in (Figure 8A–C).

## 4 Discussion

Ovarian hyperthecosis is a complex endocrine, reproductive, and metabolic condition that affects women during their reproductive age (Jing et al. 2023). The DHEA model used in our study has been demonstrated to produce ovarian hyperthecosis. Herein, in comparison to controls, the rats exposed to DHEA had multiple ovarian cysts and higher serum levels of LH, testosterone, FSH, and circRNA (hsa‐circ‐0001577).

Various circRNAs have been found to play a key role in various diseases as a result of rapid improvements in detection technologies and computational analysis (Shi and Shang 2023). Typically, several circRNAs have been identified in ovarian hyperthecosis. Granulosa cells (GCs), cumulus cells (CCs), and follicular fluid (FF) in patients with ovarian hyperthecosis showed significantly different expression of several deregulated circRNAs when compared to the control group. According to recent studies, circRNAs may serve as biomarkers for both the diagnosis and management of ovarian hyperthecosis and may be crucial in monitoring the development of the condition.

The current study revealed a significant elevation in circRNA (hsa‐circ‐0001577) post DHEA administration in the disease model. In contrast, treatment with *A. spinosus* modulated this deviated circRNA with the superiority of the high‐dose regimen. ovarian hyperthecosis revealed Granulosa cells (GC) malfunction, aberrant folliculogenesis, and ovulation disorders as a typical symptoms (Escobar‐Morreale 2018). CircRNAs can control GC function and folliculogenesis in both pathological and diseased conditions, according to new research. GCs have been shown to express CircRNAs in a stage‐specific manner during follicular development. CircRNAs function as miRNA sponges to regulate various reproductive pathways, including progesterone‐mediated oocyte maturation, oocyte meiosis, and Gonadotropin‐Releasing Hormone (GnRH) signaling (Shen et al. 2019). Additionally, it has been proposed that circRNAs play a role in the genesis and quality of oocytes. Cao et al. (2019) reported that circARMC4 knockdown dramatically disrupted chromosome alignment during porcine oocyte meiotic maturation, causing defects in porcine embryo development and that Granulosa cells revealed upregulated: hsa_circ_0001577. Thus, these circRNAs could influence ovarian function and serve as potential indicators for the destruction of the follicular environment.

Follicle formation and atresia are primarily caused by the balance between GC proliferation and apoptosis (Dompe et al. 2021). By acting as miRNA in ovarian hyperthecosis, altered expression of circRNAs can alter the level of this balance. Numerous circRNAs have been investigated to ascertain their roles in ovarian hyperthecosis's cell cycle, apoptosis, and GC proliferation (Duan et al. 2021).

The clinical features of patients with ovarian hyperthecosis are linked to the dysregulation of many circRNAs, which have been identified as potential diagnostic biomarkers for this disorder. Hsa_circ_0097636 was one of the circRNAs that was discovered to be down‐regulated in CCs and to possess good diagnostic efficacy in individuals with ovarian hyperthecosis. Four circRNAs—hsa_circ_0085997, hsa_circ_0075692, hsa‐circ‐0001577, and hsa_circ_0075691—were shown to be dysregulated in ovarian hyperthecosis and to exhibit potential biomarker action (Huang et al. 2020). These four circRNAs have not before been described in other disorders, indicating that their specificity and efficacy make them suitable biomarkers for ovarian hyperthecosis.

The current study revealed a significant elevation in LH and testosterone along with an increase in FSH post DHEA administration; meanwhile, treatment with *A. spinosus* EtOH extract modulated these dysregulated hormones with the superiority of the high dose regimen, which may be due to enhanced ovarian folliculogenesis. Increased plasma levels of androgen and LH were the most consistent hormonal feature of rats with ovarian hyperthecosis (Abbott et al. 2022). By promoting follicular development and maturation, androgens, such as DHEA, testosterone, dihydrotestosterone, and androstenedione play a crucial role in ovulation (Walters 2015). However, in ovarian hyperthecosis, too much testosterone leads to metabolic and ovulatory failure, indicating the hyperandrogenism status in the ovarian hyperthecosis condition (Rosenfield 2003). Excessive testosterone was linked to the pathophysiology of ovarian hyperthecosis in a prior study, and lowering these high testosterone levels may help treat ovarian hyperthecosis diseases (Abbott et al. 2022; Motta 2010). *A. spinosus* treatment showed a decrease in testosterone level that may reflect diminished androgen biosynthesis in the ovary.

Oxidative stress (OS) is the key contributor to the development and progression of ovarian hyperthecosis and its associated complications aside from hormonal imbalance (Mancini et al. 2021). Excessive production of reactive oxygen species (ROS) which creates an imbalance in normal cells by affecting the endogenous antioxidant defense, is termed oxidative stress (Burton and Jauniaux 2011). An imbalance between the body's capacity to detoxify and neutralize reactive oxygen species and their production contributes to oxidative stress. Numerous disorders of the reproductive system, such as ovarian hyperthecosis, endometriosis, preeclampsia, and infertility, may be influenced by oxidative stress. It has been reported that oxidative stress and ovarian hyperthecosis are correlated. Mitochondrial mutations contribute to the metabolic and hormonal dysregulation observed in ovarian hyperthecosis by impairing oxidative phosphorylation, reducing ATP synthesis, and increasing ROS production. Ovarian follicles are adversely affected by OS, which also interferes with normal follicular maturation and development. Overexposure to ROS can harm granulosa cells and oocytes inside follicles, lowering their quality and lowering fertility. Oxidative stress can result from hyperandrogenism's promotion of inflammation and insulin resistance, both of which adversely raise ROS production (Zeber‐Lubecka et al. 2023).

Accordingly, in the current study, a significant elevation was observed in the oxidative stress biomarker MDA and a reduction in the antioxidant biomarker GSH post DHEA administration; meanwhile, treatment with *A. spinosus* modulated these deviated biomarkers, with the superiority of the high dose regimen confirmed to exert antioxidant activity. Acute stress causes the blood to release high amounts of DHEA, which can affect ovarian, endocrine, and metabolic processes. According to studies, DHEA induces oxidative stress and inflammation in rat models, which leads to the development of large follicular cysts in the ovaries (Sabuncu et al. 2001). The group administered *A. spinosus* had significantly increased (*p* < 0.05) antioxidant enzyme activity (GSH) as compared with the DHEA group, thereby increasing the intracellular scavenger activity. Glutathione, a tripeptide, is an essential antioxidant in the ovary and acts as an intracellular scavenger for free radicals. This finding is consistent with Sabuncu et al. (2001) study, which reported that GSH levels were considerably lower in the ovarian hyperthecosis group than in the control group and suggested that the lower levels of GSH may have been linked to insulin resistance.

Concerning ovarian histology, induction of ovarian hyperthecosis resulted in a significant increase in the number of cystic follicles compared to the control group. These events can arise from hyperandrogenism, which leads to the formation of cystic follicles, concurrent with a decrease in the number of normal follicles (Abruzzese et al. 2022). PCOS results when ovaries produce too many androgen hormones, and reproductive hormonal imbalance results. Because of this, individuals with PCOS frequently experience irregular ovulation, missed periods, and irregular menstrual cycles. When ovulation is absent, tiny follicle cysts may be noticed on the ovaries. One of the most frequent reasons why women become infertile is PCOS. This may make other medical disorders more likely. Based on the symptoms and desire to conceive, healthcare providers can treat PCOS. Our findings are consistent with those of the other studies (Khani et al. 2021; Olaniyan et al. 2020). While the group treated with *A. spinosus* shows a normal ovarian histological architecture and a decreased number of cystic follicles, indicating the protective effect of the extract against the hormonal and ovarian morphology disturbances associated with ovarian hyperthecosis.

All the significant ovarian hyperthecosis mitigation of the *A. spinosus* EtOH extract might be attributed to its high content of long‐chain fatty acids (LCFAs), phenolic acids, flavonoids, and triterpene saponins. Long‐chain fatty acids (LCFAs) have been shown to be essential for de‐regulation of LH, FSH, and testosterone (Nagy et al. 2024). Another study also reported that LCFAs suppress the translation of FSHβ, whilst increasing the levels of LHβ and mRNA in rats (Garrel et al. 2014). Furthermore, it has been shown that phenolic compounds, particularly phenolic acids and flavonoids, possess substantial anti‐inflammatory and antioxidant properties, which successfully mitigate polycystic ovary (Hussain et al. 2022). According to Platzer et al. (2022), phenolic acids and flavonoids possess a highly conjugated structure within hydroxyl groups and aromatic skeleton characteristics that make them effective scavengers of damaging radicals as well as reactive oxygen species (ROS). They are able to prevent oxidative damage to lipids, proteins, and DNA and thus reduce inflammation in tissues by suppressing intracellular oxidative stress or negating ROS (Zhang and Tsao 2016). Furthermore, these components' antioxidant properties, interference with oxidative stress signaling pathways, and molecular‐level suppression of pro‐inflammatory mediator signaling transduction mechanisms and cellular inflammatory pathways all contribute to their capacity to reduce inflammation and, in turn, oxidative damage to tissues (Rudrapal et al. 2022).

Long‐chain fatty acids, as the main components of this plant, played a significant role in modulating ovarian hyperthecosis by influencing the hormonal balance, particularly the regulation of LH, FSH, and testosterone (Nagy et al. 2024). Long‐chain fatty acids were involved in cellular signaling pathways that can impact the function of granulosa and theca cells within the ovaries. These fatty acids interacted with G‐protein‐coupled receptors, like GPR40 and GPR120, activating intracellular cascades that regulate the release of gonadotropins and sex hormones (Liou et al. 2011). In ovarian hyperthecosis, where there is excessive androgen production, long‐chain fatty acids might help restore hormonal balance by reducing the hypersecretion of LH, which in turn diminishes ovarian testosterone production (Rosenfield and Ehrmann 2016). Additionally, long‐chain fatty acids could influence the sensitivity of the hypothalamic–pituitary–gonadal axis, thereby promoting more normal levels of FSH and LH, potentially improving follicular development and reducing the pathologic hyperandrogenism associated with the condition (Das and Kumar 2018).

This study utilized a comprehensive serum metabolomics approach to explore metabolic changes in the ovarian hyperthecosis group, control group, and two groups treated with different doses of *A. spinosus* . The OPLS‐DA model showed clear differentiation between the different groups. The analysis of the ovarian hyperthecosis group revealed a distinct metabolic profile, significantly linked through both multivariate and univariate statistical methods. A total of 22 metabolites, including lipids, amino acids, carbohydrates, and organic acids, were annotated as key factors distinguishing ovarian hyperthecosis, control, and *A. spinosus* ‐treated groups. These biomarkers further support the strong relationship between the development and treatment of ovarian hyperthecosis and the various underlying factors and mechanisms, highlighting three main metabolic pathways: tryptophan metabolism, nicotinate and nicotinamide metabolism, and arginine biosynthesis, as detailed in the next subsections.

### 4.1 Tryptophan Metabolism

Several metabolomic studies have suggested an imbalance in amino acid metabolism in ovarian disorders, particularly the notable increase in levels of aromatic amino acids, such as tryptophan, phenylalanine, and tyrosine (Buszewska‐Forajta et al. 2019). Tryptophan, an essential amino acid, is primarily metabolized via the kynurenine pathway and the serotonin pathway, as demonstrated in the flow chart (Figure 8A). Under normal conditions, over 95% of tryptophan is processed through the kynurenine pathway, with the remaining portion converted into serotonin (Oxenkrug 2010).

The changes in metabolite levels and the ratio between upstream and downstream metabolites in the tryptophan pathway are illustrated in Figure 8A. In the ovarian hyperthecosis group, the plasma concentrations of tryptophan and its metabolites, such as anthranilic acid and 5‐hydroxyindole acetate, were all increased as revealed from box plots, indicating an abnormal activation of the tryptophan catabolism pathway. Our results are in accordance with a previous report that the tryptophan‐kynurenine pathway was disrupted in women with ovarian disorders, leading to significantly increased levels of tryptophan, serotonin, kynurenine, kynurenic acid, and quinolinic acid (Wang et al. 2022). Tryptophan is converted into l‐kynurenine by indoleamine‐2,3‐dioxygenase (IDO). It has been suggested that the kynurenine/tryptophan ratio can indicate IDO activity. An increased kynurenine/tryptophan ratio implies enhanced IDO activity in ovarian disorders. Previous research has shown that IDO activity can be triggered by chronic low‐grade inflammation and negative emotions. Based on this, we hypothesized that the rise in IDO activity might be due to elevated pro‐inflammatory cytokines, such as C‐reactive protein (CRP) (Forrest et al. 2004). A recent study demonstrated that IDO activity was negatively correlated with hormone receptor activity, suggesting that IDO dysfunction could perturb hormone metabolism (Onesti et al. 2019). Interestingly, *A. spinosus* treatment significantly reduced elevated levels of key markers tryptophan and its metabolites, such as anthranilic acid and 5‐hydroxyindole acetate (Figure 8A).

### 4.2 Nicotinate and Nicotinamide Metabolism

The metabolism of nicotinate and nicotinamide has also been explored in obstetrics and gynecology. An animal study demonstrated that varying concentrations of niacin in the medium could influence the maturation quality of in vitro embryo production embryos and affect the overall tolerance of bovine oocytes to vitrification (Kafi et al. 2019). Down regulation of the nicotinate and nicotinamide metabolism pathway has been previously reported in polycystic ovarian syndrome‐like in F1 offspring mice (Kil et al. 2019). The dysregulation of that catabolic pathway can lead to elevated levels of nicotinamide (NAM). High concentrations of NAM exert adverse effects on ovarian and reproductive outcomes via disrupting cumulus expansion, increasing the incidence of spindle abnormalities, reducing the number of blastocyst cells, and adversely affecting the oocyte maturation (Ren et al. 2024). Confirming those results, Figure 8B demonstrated upstreaming in nicotine and nicotinamide (NAM) serum levels in the ovarian hyperthecosis group compared to the normal group as shown in the box plot. Evidence for *A. spinosus* effectiveness is manifested by the restoration of metabolic biomarker serum levels to their normal levels post (Figure 4B).

### 4.3 Arginine Biosynthesis Pathway

Ovarian disorders are associated with impairment in arginine biosynthesis. For instance, Öztan et al. attributed cardiovascular outcomes of polycystic ovary to methylarginine and its metabolites such as L‐arginine and L‐citrulline. This is mainly due to the involvement of methylated arginine in endothelial dysfunction, inflammation, and atherosclerosis pathophysiology. Consequently, L‐arginine and L‐citrulline are posed as promising biomarkers in clinical investigations of cardiovascular complications of polycystic ovary syndrome (Oztan et al. 2021).

Our results came in agreement with previous reports, with an elevated level of L‐citrulline detected in the serum of the ovarian hyperthecosis group (Figure 7C). The elevated serum levels of arginine, citrulline, homoarginine, and total methylarginine in the diseased group compared to the control group were possibly owed to a compensatory increase in arginine levels, the precursor molecule, to counteract endothelial dysfunction and promote nitric oxide synthesis (Oztan et al. 2021). Elci et al. (2017) also found that serum methylarginine levels were significantly higher in the diseased group, both in obese and nonobese individuals, compared to the control group. Eventually, *A. spinosus* managed to restore L‐citrulline partially to normal levels, though less evident as observed in other pathway markers previously discussed.

## 5 Conclusions

In summary, the present study employed a multiplex approach including serum metabolomics to identify 22 differential metabolic biomarkers and revealed three metabolic pathways perturbed in ovarian hyperthecosis that were restored by *A. spinosus* treatment. The results indicate that *A. spinosus* may play a role in the management of ovarian hyperthecosis by regulating circRNA (hsa‐circ‐0001577) and key pathways, including those related to tryptophan metabolism, nicotinate and nicotinamide metabolism, and arginine biosynthesis. UHPLC‐ESI‐Orbitrap‐MS profiling uncovered 72 key metabolites—primarily phenolics, triterpenoidal saponins, and pyridines—that may act alone or in synergy to manage ovarian hyperthecosis by modulating circRNA (hsa‐circ‐0001577), hormonal imbalance, and metabolomic biomarkers. Identification of the exact phytochemicals behind such effects in *A. spinosus* should follow for potential inclusion in nutraceuticals to be used for the management of ovarian hyperthecosis.

## Author Contributions

Conceptualization, N.M.A., M.A.F., and A.I.E.; methodology, N.M.A., M.O.K., A.S.A.‐E., I.E.S., R.S.I., A‐E.G.E.‐G., S.M.A., M.A.F., and A.I.E.; formal analysis, A.S.A.‐E., I.E.S., A‐E.G.E.‐G., M.A.F., and A.I.E.; investigation, N.M.A., M.O.K., A.S.A.‐E., I.E.S., R.S.I., M.A.F., and A.I.E.; resources, M.A.F., and A.I.E.; data curation, M.A.F., and A.I.E.; writing – original draft preparation, N.M.A., M.O.K., A.S.A.‐E., I.E.S., R.S.I., S.M.A., M.A.F., and A.I.E.; writing – review and editing, N.M.A., M.O.K., A.S.A.‐E., I.E.S., R.S.I., A‐E.G.E.‐G., S.M.A., T.E., M.A.F., and A.I.E.; funding acquisition, T.E., and A.I.E. All authors have read and agreed to the published version of the manuscript.

## Ethics Statement

Animals were provided with access to water and a typical diet. All animal care and treatment techniques closely follow the ethical protocols and policies established by the Animal Care and Use Committee of the National Research Center and the US National Institutes of Health, as per the approval number 04481223.

## Conflicts of Interest

The authors declare no conflicts of interest.

## Supporting information


Data S1.

## Data Availability

Data is contained within the article.
