# Supplementary material for: Amaranthus spinosus Linn. Extract as an Innovative Strategy to Regulate Biomarkers for Ovarian Hyperthecosis via Circular RNA (hsa‐circ‐0001577): Evidence From Biochemical, Metabolomics, Histological, and Phytochemical Profiling
Source: Food Sci Nutr. 2025 May 19;13(5):e70314. doi: 10.1002/fsn3.70314 (PMC12091212; doi:10.1002/fsn3.70314)
Supplement: Supplementary file 1 — Data S1. [file FSN3-13-e70314-s001.docx]

**Supporting data**

***Amaranthus spinosus Linn*. extract as an innovative strategy to regulate biomarkers for ovarian hyperthecosis *via* circular RNA (hsa-circ-0001577): Evidence from biochemical, metabolomics, histological, and phytochemical profiling**

**Naglaa M. Ammar^1^, Mai O. Kadry^1^, Asmaa S. Abd Elkarim^2^, Reham S. Ibrahim^3^, Ibrahim E. Sallam^4^, Abd El-Nasser G. El Gendy^5^, Sherif M. Afifi^6^,** **Tuba Esatbeyoglu^7, *^,** [**Mohamed A. Farag**](https://sciprofiles.com/profile/247941)**^8^, Abdelsamed I. Elshamy^9,^ ***

^1^ Therapeutic Chemistry Department, National Research Centre, Dokki, Giza 12622, Egypt

^2^ Chemistry of Tanning Materials and Leather Technology Department, National Research Centre, Dokki, Giza 12622, Egypt

^3^ Department of Pharmacognosy, Faculty of Pharmacy, Alexandria University, Egypt

^4^ Pharmacognosy Department, College of Pharmacy, October University for Modern Sciences and Arts (MSA), 6th of October City 12566, Egypt

^5^ Medicinal and Aromatic Plants Research Department, National Research Centre, Dokki, Giza 12622, Egypt

^6^ Department for Life Quality Studies, Rimini Campus, University of Bologna, Corso d’Augusto 237, 47921 Rimini, Italy

^7^ Department of Molecular Food Chemistry and Food Development, Institute of Food and One Health, Gottfried Wilhelm Leibniz University Hannover, Am Kleinen Felde 30, 30167 Hannover, Germany

^8^ Pharmacognosy Department, Faculty of Pharmacy, Cairo University, Kasr el Aini St., Cairo 11562, Egypt

^9^ Department of Natural Compounds Chemistry, National Research Centre, Dokki, Giza 12622, Egypt

***** Correspondence: [esatbeyoglu@foh.uni-hannover.de](mailto:esatbeyoglu@foh.uni-hannover.de) (T.E.); [elshamynrc@yahoo.com](mailto:elshamynrc@yahoo.com) (A.I.E.)

******

**Figure (S1):** Representative base peak chromatogram of *Amaranthus spinosa* in negative ionization mode

**Table (1S)**: Key differential metabolites of serum samples of control (C), ovarian hyperthecosis-diseased (D) and dose-based *A. spinosus*- treated (T) groups arranged from lowest to highest P-value

| **Differential metabolites name** | **M.wt.** | **Molecular formula** | **HMDB ID** | **P-value** | **VIP** | **Trend  (C-D)** | **Trend  (D-T)** |
| --- | --- | --- | --- | --- | --- | --- | --- |
| Heptadecanoic acid | 270.45 | C_17_H_34_O_2_ | HMDB0002259 | 2.26E-08 | 1.39712 | ↑ | ↓ |
| Docosahexaenoic acid | 328.48 | C_22_H_32_O_2_ | HMDB0002183 | 2.17E-06 | 1.13833 | ↑ | ↓ |
| Phenylpropionic acid | 150.17 | C_9_H_10_O_2_ | HMDB0011743 | 9.87E-06 | 1.34343 | ↓ | ↑ |
| 2-Ketoisocaproic acid | 130.14 | C_6_H_10_O_3_ | HMDB0000408 | 3.01E-05 | 1.04378 | ↑ | ↓ |
| 5-Hydroxyindoleactate | 191.18 | C_10_H_9_NO_3_ | HMDB0000763 | 5.03E-05 | 1.07658 | ↑ | ↓ |
| Palmitoleic acid (C16:1n7) | 254.4 | C_16_H_30_O_2_ | HMDB0003229 | 6.31E-05 | 1.32185 | ↑ | ↓ |
| L-Tryptophan | 204.22 | C_11_H_12_N_2_O_2_ | HMDB0000929 | 8.72E-05 | 1.07504 | ↑ | ↓ |
| 1-Monoolein | 356.53 | C_21_H_40_O_4_ | HMDB0011567 | 0.000117 | 1.31349 | ↑ | ↓ |
| 1-Monostearin | 358.55 | C_21_H_42_O_4_ | HMDB0011131 | 0.000178 | 1.06737 | ↑ | ↓ |
| Glucose-1-phosphate | 260.13 | C_6_H_13_O_9_P | HMDB0001586 | 0.000228 | 1.20828 | ↑ | ↓ |
| Anthranilic acid | 137.13 | C_7_H_7_NO_2_ | HMDB0001123 | 0.000446 | 1.34062 | ↑ | ↓ |
| Nicotinamide | 122.12 | C_6_H_6_N_2_O | HMDB0001406 | 0.00061 | 1.28544 | ↑ | ↓ |
| 5-Oxoproline | 129.11 | C_5_H_7_NO_3_ | HMDB0000267 | 0.001506 | 1.27675 | ↑ | ↓ |
| Myristic acid | 228.37 | C14H28O2 | HMDB0000806 | 0.0017 | 1.3026 | ↑ | ↓ |
| Citrulline | 175.18 | C_6_H_13_N_3_O_3_ | HMDB0000904 | 0.002925 | 1.07938 | ↑ | ↓ |
| Arabitol | 152.14 | C_5_H_12_O_5_ | HMDB0001851 | 0.009505 | 1.09092 | ↓ | ↑ |
| Oleamide | 281.47 | C_18_H_35_NO | HMDB0002117 | 0.011591 | 1.09395 | ↑ | ↓ |
| D-(-)-Ribose | 150.13 | C_5_H_10_O_5_ | HMDB0000621 | 0.0184 | 1.19223 | ↓ | ↑ |
| Glyceric acid | 106.07 | C_3_H_6_O_4_ | HMDB0000139 | 0.021746 | 1.08914 | ↓ | ↑ |
| alpha-Methylserine | 119.11 | C_4_H_9_NO_3_ | HMDB0341365 | 0.022741 | 1.06248 | ↑ | ↓ |
| Nicotinic acid | 123.1 | C_6_H_5_NO_2_ | HMDB0001488 | 0.03119 | 1.11742 | ↑ | ↓ |
| D-Asparagine | 132.11 | C_4_H_8_N_2_O_3_ | HMDB0033780 | 0.037576 | 1.02547 | ↓ | ↑ |
